# Degradation of Herpes Simplex Virus-1 Viral miRNA H11 by Vaccinia Virus Protein VP55 Attenuates Viral Replication

**DOI:** 10.3389/fmicb.2020.00717

**Published:** 2020-04-23

**Authors:** Weixuan Zou, Xusha Zhou, Lei Wang, Grace Guoying Zhou, Xiaoqing Chen

**Affiliations:** ^1^School of Basic Medical Sciences, Guangzhou Medical University, Guangzhou, China; ^2^Shenzhen International Institute for Biomedical Research, Shenzhen, China

**Keywords:** miR-H11, VP55, DNA synthesis, replication, herpes simplex virus-1

## Abstract

Among 29 distinct miRNAs expressed by the herpes simplex virus-1 (HSV-1) during lytic infection, miR-H11, together with miR-H1 to miR-H8 are reported to locate in the RNA-induced silencing complex (RISC). miR-H11 is encoded within viral origins of replication and lies entirely within the origins of replication. However, the roles of this miRNA derived from lytic infection with HSV-1 remain unclear. Using the advantage of vaccinia virus protein VP55 (VP55)-mediated degradation of miRNAs, we constructed a recombinant virus expressing VP55 (R5502) to demonstrate that: (1) accumulation of miR-H11 from R5502 was reduced by 540-fold versus that in cells infected with wild-type HSV-1, but miR-H1 to miR-H8 which also located in the RISC were not reduced significantly from R5502 compare with wild-type HSV-1; (2) downregulation of miR-H11 from R5502 infected cells results in markedly lower viral DNA synthesis compared with wild-type HSV-1; and (3) downregulation of miR-H11 also restricted viral spreading, and resulted in low accumulation of representative viral proteins and viral yields. The findings were confirmed through either using of a miR-H11 inhibitor or pre-transfection of a plasmid expressing VP55. These data suggest that miR-H11 plays a currently unidentified role in maintaining sufficient viral DNA synthesis during the course of viral infection.

## Introduction

miRNAs are derived from primary transcript (pri-miRNA) and modulated at the levels of transcription and processing (Johnson et al., 2003; Hagan et al., 2009; Heo et al., 2009; Liu and Liu, 2011). Pri-miRNA is recognized by a nuclear microprocessor complex, which includes the RNase III enzyme Drosha, and cleaved into a precursor miRNA (pre-miRNA; Siomi and Siomi, 2010). The pre-miRNA is subsequently cleaved by Dicer to generate a duplex RNA of ∼22 nt (Grishok et al., 2001; Hutvagner et al., 2001; Ketting et al., 2001), which is subsequently loaded into an RNA-induced silencing complex (RISC; Czech and Hannon, 2011) to produce mature miRNA (Grishok et al., 2001; Hutvagner et al., 2001; Ketting et al., 2001). Vaccinia virus protein VP55 (VP55) has been shown to be both necessary and sufficient for the tailing of RISC-associated host miRNAs (Backes et al., 2012). VP55 adds non-templated adenosines specifically to the miRNAs, which are associated with the RISC; hence, it results in the rapid degradation of those miRNAs (Backes et al., 2012).

Among 29 discovered miRNAs expressed by the herpes simplex virus-1 (HSV-1) during lytic infection with HSV-1, miR-H11 is located in the RISC. Other miRNAs located in the RISC are miR-H1 to miR-H8 (Flores et al., 2013). miR-H11 is encoded within the viral origins of replication (OriL; Jurak et al., 2014; Du et al., 2015) and lies entirely within the OriL (Jurak et al., 2014; Du et al., 2015). The 65 nucleotides at the 5′ terminus of the H11 precursor are complementary to the 65 nucleotides at its 3′ terminus. H11 represents the highest increase observed for the interval between 1 and 12 h after infection with HSV-1 (F) in HEp-2 cells. However, it is not detected in ganglia harboring latent virus or in ganglia incubated for 24 h in medium containing anti-nerve growth factor antibody (Du et al., 2015).

In this study, we constructed an HSV-1 recombinant virus expressing VP55 (R5502) to assess the impact of miR-H11 loss on virus replication. Our data demonstrated that accumulation of miR-H11 was reduced by 540-fold compared with that in wide-type cells infected with HSV-1. In turn, downregulation of miR-H11 resulted in lower viral DNA synthesis, restriction of viral spreading, and low viral yields.

## Materials and Methods

### Cell Lines and Virus

HEp-2 and Vero cells were obtained from the American Type Culture Collection and cultured in Dulbecco’s Modified Eagle Medium (DMEM; high glucose content) supplemented with 5% (v/v) fetal bovine serum (FBS), or 5% (v/v) newborn calf serum (NBCS), respectively. HSV-1(F), the prototype HSV-1 strain used in this laboratory, was propagated and titrated using Vero cells.

### Antibodies

Antibodies against ICP8 (Rumbaugh Goodwin Institute for Cancer Research, Inc.), ICP0 (Cat No. ab6513; Abcam), ICP4 (Cat No. ab6514; Abcam), ICP27, VP16, and US11 have been described elsewhere (Ackermann et al., 1984; McKnight et al., 1987; Roller and Roizman, 1992). Antibodies against ICP22, VP22, and VP16 were kind gifts of Bernard Roizman (The University of Chicago, United States). Additional antibodies used in this study were anti-green fluorescent protein (anti-GFP) monoclonal antibody (Cat No. KM8009; Sungene Biotech) and anti-glyceraldehyde-3-phosphate dehydrogenase (anti-GAPDH; Cat No.KM9002; Sungene Biotech).

### miRNA Inhibitors

Non-target (NT) inhibitor and miR-H6-5p, H3-3p, and H11 inhibitors were designed and purchased from GenePharma. The sequences were as follows:

NT inhibitor: 5′-CAGUACUUUUGUGUAGUACAA-3′,

miR-H6-5p inhibitor: 5′-UACACCCCCCUGCCUUCCACC-3′,

miR-H3-3p inhibitor: 5′-GUCCCAACCGCACAGUCCCAG-3′,

miR-H11 inhibitor: 5′-GCGUUCGCACUUUGUCCUAA-3′.

### Construction of the Plasmid

The plasmid enhanced green fluorescent protein-VP55 (pEGFP-VP55) (p5502) containing VP55 in fusion with EGFP was a kind gift of Benjamin R. (Mount Sinai School of Medicine, United States). The control plasmid pGFP02 was designed to insert a stop codon (TGA) immediately after the VP55 ATG start codon.

### Construction of Recombinant Viruses

We constructed the VP55 recombinant virus (R5502) and control virus (RGFP02). The gene encoding VP55 or GFP was inserted into the genes encoding UL3 and UL4 under the cytomegalovirus promoter, respectively. The strategy for the construction of the virus has been previously reported (Ren et al., 2019).

### Transfection of Cells

HEp-2 cells were seeded in 12-well plates the day prior to transfection in DMEM containing 5% FBS. The following day, the plasmids or HSV-1 miRNA inhibitors described above were transfected into HEp-2 cells. Lipofectamine 2000 (Invitrogen) was used for transfection according to the protocol provided by the manufacturer. At 24 h post transfection, the cells were infected with 0.1 or 1 plaque-forming unit (PFU) of HSV-1(F) per cell, harvested at an indicated time point post infection or at 48 h post transfection, and lysed according to the protocol for the subsequent analyses.

### Immunoblotting Assays

Cell lysates were harvested and lysed with a radioimmunoprecipitation assay lysis buffer (Beyotime) supplemented with 1 mM protease inhibitor phenyl methyl sulfonyl fluoride (Beyotime) and heat denatured, separated using sodium dodecyl sulfate-polyacrylamide gel electrophoresis, and transferred to polyvinylidene difluoride membranes (Millipore). The proteins were detected through incubation with an appropriate primary antibody, followed by incubation with horseradish peroxidase-conjugated secondary antibody (Invitrogen). Visualization was performed using the enhanced chemiluminescence reagent (Pierce) and film exposure, or the capture of images using the ChemiDoc Touch Imaging System (Bio-Rad) and processed using the Image Lab software. The densities of corresponding bands were quantified using the ImageJ software.

### Virus Titration

HEp-2 cells were seeded in a six-well plate at densities of 1 × 10^6^ cells per well. The cells were subsequently exposed to 0.01 PFU of HSV-1(F), VP55 recombinant virus (R5502) and control virus (RGFP02), or 0.1 PFU of HSV-1(F) per cell at 24 h post plasmid transfection. The cells were harvested at 3, 6, 12, 24, 48 and 72 h, or 3, 6, 12 and 24 h post infection. Viral progeny was titrated using Vero cells following three freeze-thaw cycles.

### Plaque Assay

Vero cells seeded in six-well plates were exposed to 0.001 PFU of HSV-1(F), R5502, and RGFP02 per cell for 2 h and maintained in 199V medium (Gibco) supplemented with 1% FBS for 48 h. The cells were fixed with 4% (w/v) of paraformaldehyde for 30 min, rinsed thrice with phosphate-buffered saline, and stained with Giemsa stain for 30 min. The images were captured using an inverted Leica microscope.

### Viral miRNA Deep Sequencing

HEp-2 cells were infected with 10 PFU of HSV-1(F) and VP55 recombinant virus (R5502) per cell. The cells were harvested 24 h post infection. Small RNAs were isolated and subjected to high-throughput sequencing by Capital Bio Technology to identify HSV-1- and R5502-derived miRNAs.

### HSV Genome Labeling and Click Chemistry Study

HEp-2 cells grown on glass coverslips at densities of 1.5 × 10^4^ cells overnight were serum-starved in DMEM containing 0.25% FBS for 24 h to arrest cells at the G_0_ stage (Ren et al., 2016). The cells were subsequently infected with HSV-1 (F), R5502, or RGFP02 at 10 PFU per cell for 1 h, the medium was replaced with fresh DMEM containing 1% FBS and 10 μM 5-ethynyl-2′-deoxyuridine (EdU), and the cells were cultured for indicated hours. EdU-labeled DNA was conjugated with Alexa Fluor 647 picolyl azide using the Click-iT Plus EdU imaging kit (Life Technologies). The nuclei were stained with 4′,6-diamidino-2-phenylindole (DAPI). The images were captured and processed using a confocal laser-scanning microscope (magnification, 40×). The EdU/GFP-positive cells were quantified using the ImageJ software.

## Results

### VP55 Significantly Downregulates Viral miRNA miR-H11

#### Construction of the VP55 Plasmid and HSV-1 Expressing the VP55 Recombinant Virus

**FIGURE 1:**
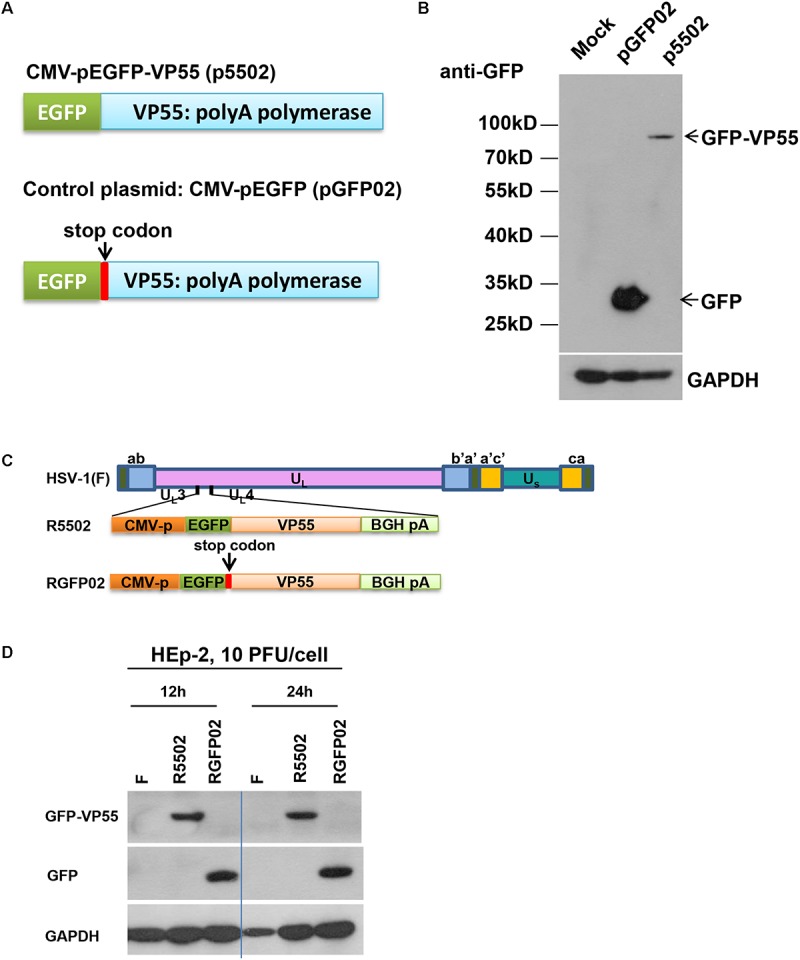
**(A)** Schematic diagram of the VP55 expression plasmid (p5502) and control plasmid (pGFP02). p5502 was designed to express VP55 in fusion with EGFP based on the pEGFP-C1 plasmid. The VP55 coding sequence was inserted in the C-terminus of EGFP. pGFP02 is the control plasmid, which was constructed through insertion of a TGA stop codon immediately after the VP55 ATG start codon. **(B)** Protein expression levels in cells transfected with the VP55 expression plasmid (p5502) and control plasmid (pGFP02). HEp-2 cells were mock-treated or transfected with 0.75 μg of pGFP02 or p5502 plasmid in a 12-well plate. The cells were harvested 48 h post transfection. Accumulations of GFP and VP55-GFP were measured as described in the “Materials and Methods” section. **(C)** Schematic representation of the parent virus HSV-1(F), VP55-expressing recombinant virus (R5502), or control recombinant virus (RGFP02). R5502, derived from the parent wild-type HSV-1(F), is a recombinant virus expressing VP55 fused with EGFP. RGFP02 is the control recombinant virus, which only expressed EGFP. All constructs were inserted into UL3 and UL4 genes, and the open reading frames (ORFs) were driven by the CMV promoter and tailed with BGH-polyA signal. **(D)** The GFP-VP55 fusion protein or GFP expressed by the recombinant virus was analyzed through infection with 10 PFU of HSV-1(F), R5502, and RGFP02 per cell. The cells were harvested at 12 and 24 h post infection. The accumulations of GFP and VP55-GFP were measured using an immunoblotting assay as described in the “Materials and Methods” section.

In this series of studies, we employed a VP55 plasmid and HSV-1 expressing the VP55 recombinant virus to assess the impact of viral miRNA loss on the infection. The structure of the plasmid which expressed EGFP-fused VP55 (p5502) and the control plasmid pGFP02 which expressed EGFP only are shown schematically in Figure 1A. In brief, the expression of VP55 was optimized using a human codon (Backes et al., 2012) and fusion was performed with an EGFP (p5502). The control plasmid (pGFP02) was constructed through insertion of a stop codon immediately after the VP55 start codon, resulting in the expression of EGFP only (Figure 1A). For the characterization of protein expression, HEp-2 cells were transfected with p5502 or pGFP02 and harvested 48 h later. Cell lysates were prepared and subjected to electrophoresis in denaturing gels, followed by incubation with an anti-GFP antibody. The detected bands were approximately 80 and 30 kDa in the p5502 and pGFP02 plasmid transfection samples, respectively (Figure 1B), consistent with the calculated molecular sizes. The effect of degradation of overall host miRNAs by VP55 was confirmed through transfection of p5502 or pGFP02 into HEp-2 cells and subsequent detection of the accumulation of host miRNAs (Let-7a, miR-93, and miR-21). The accumulation of host miRNAs in cells transfected with p5502 was reduced by approximately three-fold. In contrast, there was no reduction of these miRNAs in cells transfected with pGFP02 (Supplementary Figure S1 and Supplementary Table S1). We subsequently constructed the recombinant virus expressing EGFP-fused VP55 (R5502) and the control virus (RGFP02) expressing EGFP only (Figure 1C). The protein-coding sequences driven by the cytomegalovirus promoter were inserted into the *UL3* and *UL4* genes. The expression of GFP-fused VP55 from R5502 and GFP from RGFP02 were determined through infection of R5502 and RGFP02 in HEp-2 cells (10 PFU per cell, 12 and 24 h) and subsequent blotting of the cell lysates with anti-GFP antibody (Figure 1D).

#### Deep-Sequencing Analyses of Cells Infected With R5502 Led to the Identification of miR-H11, Which Is Markedly Downregulated by VP55

**FIGURE 2:**
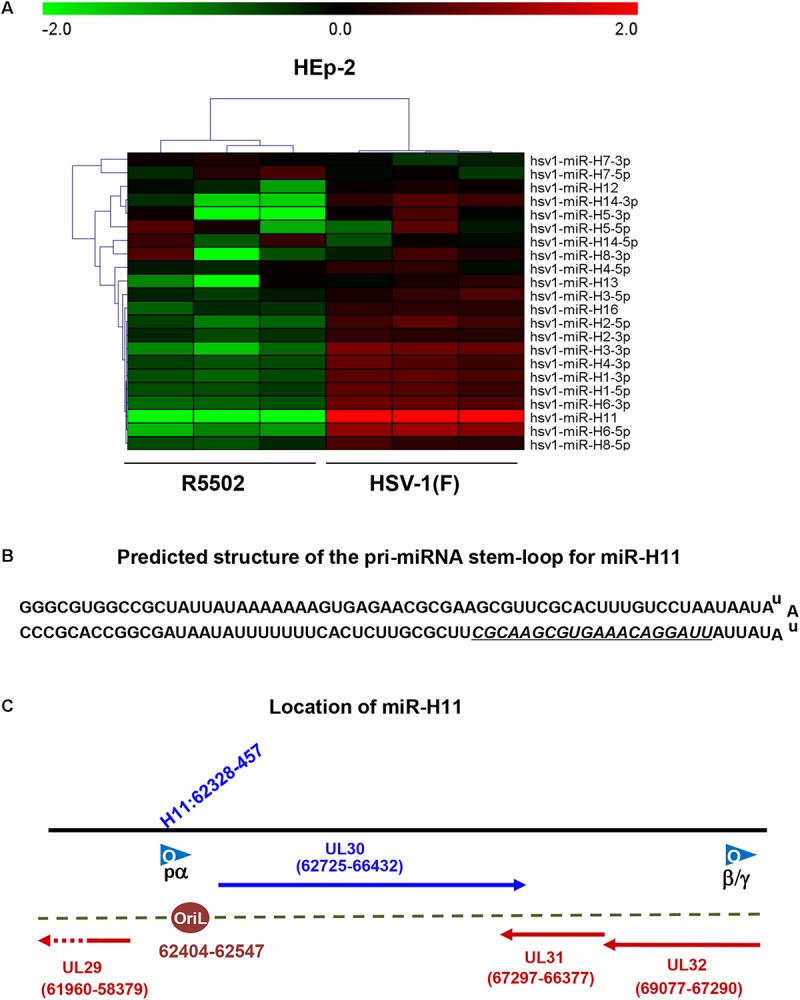
VP55 recombinant virus (R5502) displays decreased expression of most of viral miRNAs, as revealed by miRNA Deep-Seq analyses. **(A)** The heat map depicts the fold-change in the expression of viral miRNAs in HEp-2 cells infected with HSV-1(F) and R5502. HEp-2 cells were exposed to 10 PFU of HSV-1(F) or R5502 per cell for 24 h. The cells were harvested and RNA was extracted for miRNA deep-Seq analyses. Relative expression levels are depicted using different colors: red, upregulation; green, downregulation (*n* = 3). **(B)** Predicted structure of the pri-miRNA stem-loop for miR-H11, which consists of a perfect 65-nucleotide inverted repeat. Underline indicates the mature miR-H11 sequence. **(C)** The location of miR-H11 in the HSV-1(F) genome, derived from the HSV-1 origin of replication OriL.

We investigated the overall expression of viral miRNAs in HEp-2 cells infected with R5502 and HSV-1(F) by performing a microRNA deep-sequencing analysis (Figure 2A). Comparative analyses of the miRNAs profiles showed that, among all the viral miRNAs tested, three were present in significantly low amounts in R5502-infected cells (Figure 2A). The amounts of miR-H11, H3-3p, and H6-5p were reduced by 540-, 2.1-, and 2.6-fold (Table 1). As the key effector of miRNA, miR-H11 is located in the RISC (Flores et al., 2013), which may explain its high degradation by VP55.

**TABLE 1:** Viral miRNAs are reduced more than two-fold from R5502 infection versus HSV-1(F) infection.

**Cell line**	**miRNAs**	**Reduction fold**
	H3-3p	2.1
HEp-2	H6-5p	2.6
	H11	540.4

### Impact of miR-H11 Downregulation on Viral Replication

#### Downregulation of miR-H11 Results in Lower Viral DNA Synthesis

**FIGURE 3:**
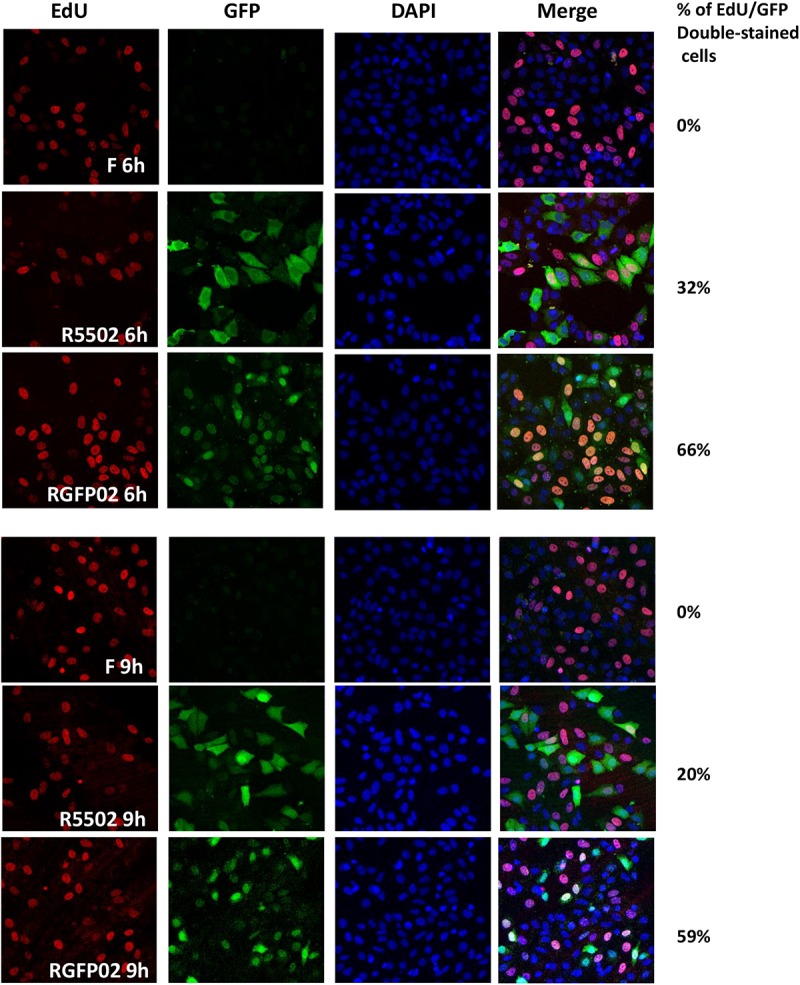
VP55 recombinant virus (R5502) displays defective viral DNA synthesis ability. Serum-starved HEp-2 cells were exposed to 10 PFU of HSV-1(F), R5502, or RGFP02 per cell. After 1 h, the inoculum was replaced with fresh medium containing EdU conjugated with Alexa Fluor 647 (red). The cells were fixed at the indicated time points and stained with DAPI (blue, for nuclei). The images were captured and processed using a confocal laser-scanning microscope (magnification, 40×). The EdU- and GFP-positive cells were quantified using the ImageJ software, and the percentage of EdU and GFP double-stained cells in GFP-stained cells were calculated.

miR-H11 is encoded within a unique sequence (Figure 2B). The 65 nucleotides at the 5′ terminus of the H11 precursor are complementary to the 65 nucleotides at its 3′ terminus (Flores et al., 2014). Interestingly, miR-H11 is encoded within the viral OriL (Figure 2C) and could be derived from previously reported transcripts that span the viral origins (Jurak et al., 2014). We used the EdU incorporation method to measure the replication of the R5502 viral genome and to investigate whether reduction of miR-H11 affects viral DNA synthesis. In principle, EdU incorporation remains scarce in serum-starved cells, whereas EdU is incorporated into newly synthesized HSV-1 genomic DNA. In brief, serum-starved HEp-2 cells were exposed to 10 PFU of HSV-1(F), R5502, or RGFP02 per cell for 1 h. The inoculum was replaced with fresh medium which contained 1% FBS and 10 μM EdU conjugated with Alexa Fluor 647. The cells were fixed at 6 and 9 h post infection and stained with DAPI. The cells infected with R5502 or RGFP02 were visualized using the GFP signal (Figure 3). Notably, there was single staining of EdU over the F, R5502- and RGFP02-infected cells due to the cells were not fully starved to synchronize the cell cycle. From each image, we have counted all the number of GFP positive cells (green) first, then counted the EdU positive-staining nucleus (red) only from GFP positive cells. In summary, at 6 h post infection, the percentage of EdU and GFP double-stained cells from cells infected with R5502 and RGFP02 was 32 and 66%, respectively. At 9 h post infection, this percentage was 20 and 59%, respectively, indicating that reduction of miR-H11 results in a defect in viral DNA synthesis.

#### Treatment of HEp-2 Cells With miR-H11 Inhibitor Prior to Infection With F Results in Decreased Accumulation of Viral Proteins

**FIGURE 4:**
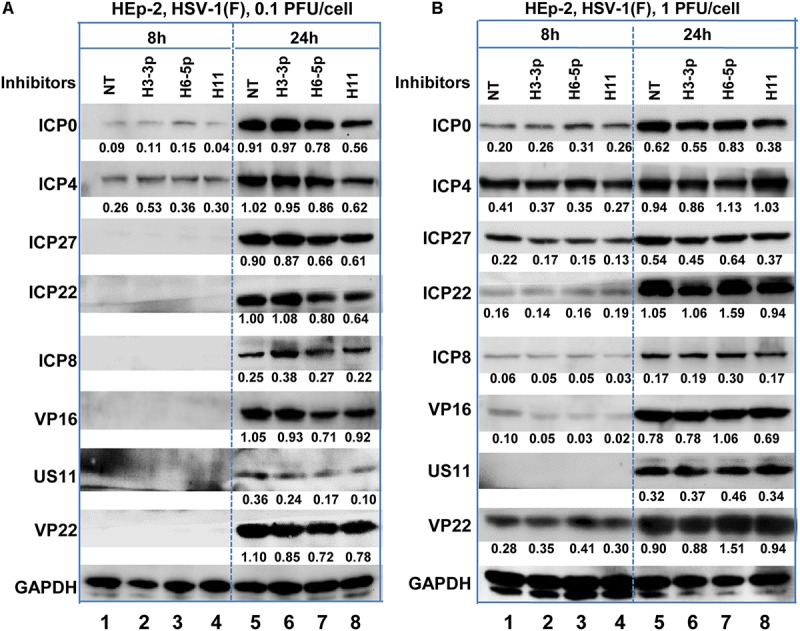
Accumulation of viral protein in HEp-2 cells transfected with viral miRNA inhibitors. Replicate HEp-2 cultures containing 2.5 × 10^5^ cells were transfected with 100 nM of non-target (NT), miR-H3-3p, H6-5p, and H11 inhibitors for 24 h, and subsequently exposed to 0.1 **(A)** or 1 PFU **(B)** of HSV-1(F) per cell for 8 and 24 h. The cells were harvested, and the proteins were electrophoretically separated using 10% denaturing gels and incubated with antibodies against ICP0, ICP4, ICP27, ICP22, ICP8, US11, VP16, VP22, or GAPDH.

According to the results of the microRNA deep-sequencing analysis shown in Figure 2A and Table 1, we subsequently investigated the role of H3-3p, H6-5p, and H11 in HSV-1 infection because technically result with R5502 could be a consequence of downregulation of H11, H3-3p or H6-5p. HEp-2 cells were transfected with inhibitors of Non-target inhibitor (NT), H3-3p, H6-5p, and H11. After 24 h, the cells were exposed to 0.1 PFU (Figure 4A) or 1 PFU (Figure 4B) of HSV-1(F) per cell. The cells were harvested at 8 or 24 h after infection and subjected to electrophoresis in denaturing gels, followed by incubation with antibodies against the α gene products ICP0, ICP4, ICP27, ICP22, antibody against ICP8 (a β gene product), or antibodies against the γ gene products VP16, US11, VP22. GAPDH served as a loading control. The densities of corresponding bands were quantified using the ImageJ software and all relative to GAPDH. In summary, at MOI of 0.1, the accumulations of ICP0, ICP4, ICP27, ICP22, Us11, VP22 are decreased by H11 inhibitor relative to NT and inhibitors of H3-3p, H6-5p at 24 h. The accumulations of these viral proteins by inhibitors of H3-3p, H6-5p are not decreased from those relative to NT (Figure 4A, lanes 5, 6, 7, 8). At MOI of 1, there are not much difference between treatment of the three inhibitors and NT treatment at 8 and 24 h (Figure 4B). So we conclude that the effect of miR-H11 inhibitor to viral replication is more significant at low multiplicity.

### Properties of the VP55 Plasmid and HSV-1 Expressing the VP55 Recombinant Virus Generated in This Study

#### VP55 Recombinant Virus (R5502) Showed Defect in Viral Protein Accumulation in HEp-2 Cells

**FIGURE 5:**
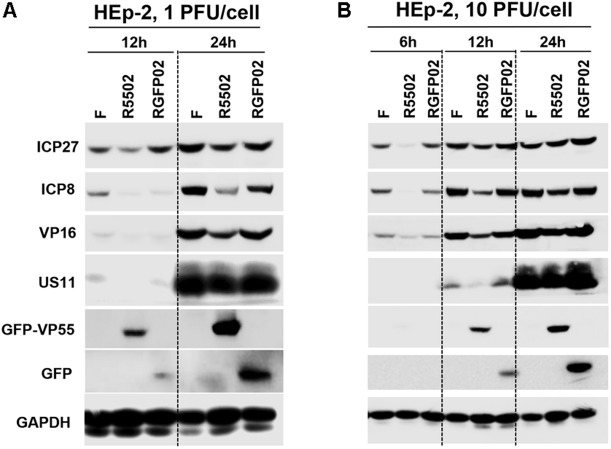
Decreased protein expression following infection with VP55-expressing recombinant virus (R5502). **(A,B)** Accumulation of R5502, RGFP02, and wild-type HSV-1(F) viral proteins in HEp-2 cells. HEp-2 cells were mock-infected or exposed to 1 PFU **(A)** or 10 PFU **(B)** of HSV-1(F), R5502, or RGFP02 per cell in a 12-well plate. The cells were harvested at the indicated time points post infection. The proteins were electrophoretically separated using 10% denaturing gels and incubated with antibodies against ICP27, ICP8, VP16, US11, GFP, or GAPDH.

In this series of experiments, replicate cultures of HEp-2 cells were exposed to 1 PFU (Figure 5A), or 10 PFU (Figure 5B) of HSV-1(F), R5502, or RGFP02 per cell. The cultures were harvested at indicated time points after infection, followed by solubilization, electrophoresis in denaturing gels, and incubation with antibodies against ICP27, ICP8, VP16, US11, GFP, and GAPDH. Of note, ICP27, ICP8, VP16, and US11 represent different kinetic classes of virus replication. GFP is a positive indicator of recombinant virus and GAPDH served as a loading control. The accumulation of ICP27, ICP8 and VP16 from R5502 infected cells at 1 PFU/cell, 12 or 24 h or 10 PFU/cell 6, 12, 24 h post infection are lower than those from either F or RGFP02 infected cells at same time points. The accumulation of US11 from R5502 infected cells at 12 h from 1 or 10 PFU/cell are also lower than what from either F or RGFP02 infected cells, at late infection, there is no difference of US11 accumulation from F, R5502 or RGFP02. There was no difference in viral protein accumulation between HSV-1(F)- and RGFP02-infected cells. Importantly, the VP55-expressing virus R5502 induced a defect in viral protein accumulation.

#### VP55 Recombinant Virus Showed Limited Spreading Ability in Vero Cells

**FIGURE 6:**
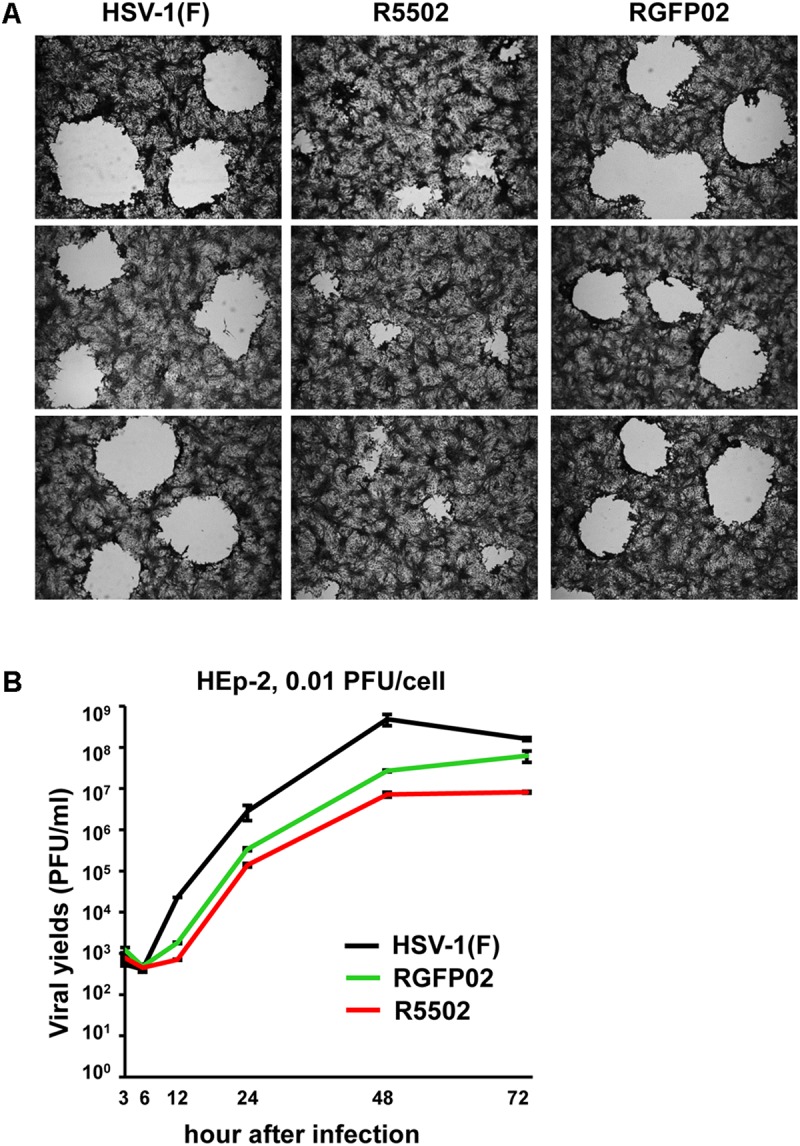
Decreased viral spreading ability and yields of the VP55 recombinant virus (R5502). **(A)** Vero cells grown in six-well plates were exposed to 0.001 PFU of HSV-1(F), R5502, or RGFP02 per cell for 2 h, and subsequently rinsed and overlaid with medium 199V. At 48 h post infection, the cells were fixed with 4% (w/v) paraformaldehyde for 30 min, stained with Giemsa stain for 30 min, and photographed at 5× magnification with the aid of an inverted microscope. **(B)** Viral yields of VP55 recombinant virus (R5502) in HEp-2 cells. HEp-2 cells were exposed to 0.01 PFU of HSV-1(F), RGFP02 or R5502 per cell. After 2 h, the inoculum was replaced with fresh medium. The virus progeny was harvested at he indicated time points and titrated using Vero cells.

Vero cells were infected with 0.001 PFU of HSV-1(F), R5502, or RGFP02 per cell. The cultures were fixed and stained with Giemsa stain at 48 h post infection, as described in the “Materials and Methods” section. Representative plaques photographed at the same magnification are shown in Figure 6A. The size of plaques formed by R5502 in Vero cells was markedly smaller than that of plaques formed in RGFP02- and HSV-1(F)-infected cells, indicating the spreading of the R5502 virus from cell to cell was markedly impaired. Figure 6B shows the yields of R5502, RGFP02 and HSV-1(F) in HEp-2 cells. In this experiment, HEp-2 cells were exposed to 0.01 PFU per cell, and virus progeny was harvested at the indicated time points and titrated in Vero cells. The results shown in Figure 6B indicate that the accumulation of virus in cells infected with R5502 was lower than those obtained from cells infected with RGFP02 and HSV-1(F) at 12, 24, 48, and 72 h post infection even though the accumulation of virus in cells infected with RGFP02 was notably lower than those obtained from cells infected with HSV-1(F). The results suggest that down-regulation of miR-H11 by R5502 negatively affects the replication of HSV-1.

#### Transient Transfection of VP55 Plasmid Results in Decreased Accumulation of Viral Proteins and Reduced Yields of Virus in HEp-2 Cells

**FIGURE 7:**
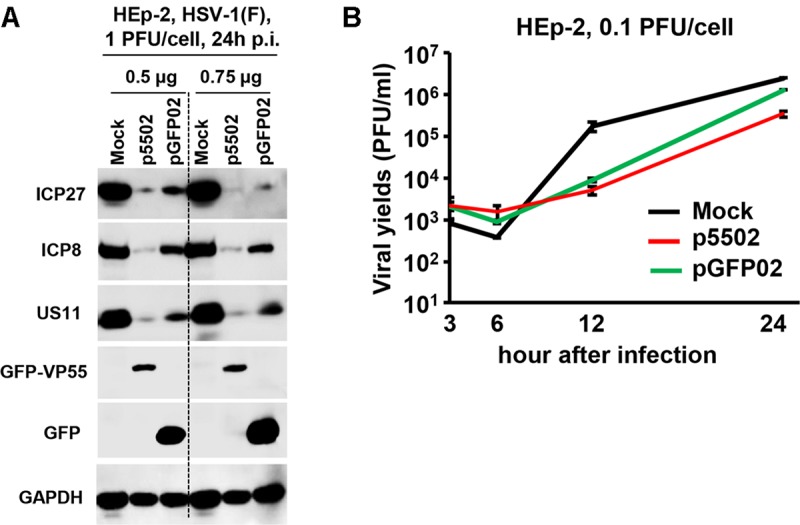
Accumulation of viral proteins and viral yields in cells mock-transfected or transfected with the VP55 expression plasmid (p5502) and control plasmid (pGFP02). **(A)** Viral protein accumulation in HEp-2 cells transfected with p5502 and pGFP02. HEp-2 cells were mock-transfected or transfected with 0.5 or 0.75 μg of p5502 or pGFP02 for 24 h, followed by exposure to 1 PFU of HSV-1(F) per cell in a 12-well-plate. The cells were harvested at 24 h post infection (p.i.). The proteins were electrophoretically separated using 10% denaturing gels and incubated with antibodies against ICP27, ICP8, US11, GFP, or GAPDH. **(B)** Viral yields in p5502- and pGFP02-transfected cells. HEp-2 cells were mock-transfected or transfected with 1.5 μg of p5502 and pGFP02 for 24 h, followed by exposure to 0.1 PFU of HSV-1(F) per cell in six-well plate. After 2 h, the inoculum was replaced with fresh medium. The virus progeny was harvested at the indicated time points and titrated using Vero cells.

Replicate cultures of HEp-2 cells were transfected with 0.5 or 0.75 μg of p5502 or pGFP02 for 24 h and subsequently exposed to 1 PFU of HSV-1(F) per cell. The cultures were harvested following infection, solubilized, subjected to electrophoresis in denaturing gels, and incubated with antibodies against ICP27, ICP8, and US11 representing different kinetic classes of viral replication. GFP is a positive indicator of plasmid transfection and GAPDH served as a loading control (Figure 7A). The results showed that the accumulation of viral proteins in cells transfected with the two different doses of p5502 was lower than that observed in cells transfected with pGFP02 (Figure 7A). Figure 7B shows the yields of HSV-1(F) in p5502- or pGFP02-transfected HEp-2 cells. In this experiment, HEp-2 cells were mock-transfected or transfected with p5502 and pGFP02 for 24 h, followed by exposure to 0.1 PFU of HSV-1(F) per cell. The virus progeny was harvested at the indicated time points and titrated using Vero cells. The data showed that at 24 h post-infection, viral yields from p5502-transfected cells were four-fold lower than those from pGFP02 and seven-fold lower than from mock-transfected cells. At 12 h of infection, both pGFP02 and p5502-transfected cells shown highly lower virus yield because of DNA transfection overall interferes HSV replication.

## Discussion

Accumulating evidence supports the hypothesis that miRNAs play roles in infection by herpes simplex viruses. This evidence suggests a model of infection, in which the production of virus and its virulent effects are tightly controlled to maximize persistence in the host and population (Umbach et al., 2008; Umbach et al., 2010; Sun and Li, 2012; Du et al., 2015; Huang et al., 2019).

Backes et al. reported that the vaccinia virus exploits the cellular miRNA pathway (Backes et al., 2012). Furthermore, they discovered that VP55 is both necessary and sufficient for miRNA polyadenylation to mediate the degradation of miRNAs in mammalian cells. This is achieved by adding non-templated adenosines specifically to the miRNAs associated with the RISC. We wished to determine the global role of HSV-1 viral miRNAs in the cellular response to viral infection. For this purpose, we generated a plasmid expressing VP55 and HSV-1 expressing the VP55 recombinant virus to rapidly eliminate viral miRNA populations. The miRNAs expressed by HSV-1 located in the RISC are miR-H1 to miR-H8 and H11 (Flores et al., 2014). Surprisingly H11, H3-3p, and H6-5p were the only three miRNAs reduced among all the viral miRNAs. Notably, the amount of H11 was reduced by 540-fold, confirming from a different angle that it is truly located in the RISC.

Unlike the other reported 29 miRNAs of HSV-1, the structure of the miR-H11 precursor is unique. This precursor is self-complementary. Its 65 nucleotides at the 5′ terminus are complementary to the 65 nucleotides at its 3′ terminus, and H11 lies entirely within the OriL (Jurak et al., 2014; Du et al., 2015). The HSV genome contains three OriL: OriL is present once in UL and OriS is present twice in the repeated C region. OriL is located between genes encoding replication proteins, ICP8 (UL29), and the catalytic subunit of polymerase (Weller and Coen, 2012). Therefore, it is relatively difficult to investigate the function of H11 through mutation without affecting the OriL to produce recombinant virus. The introduction of VP55 into HSV-1 provides us with an alternative approach. We detected markedly lower viral DNA synthesis versus wild-type HSV-1, as a result of low accumulation of viral proteins and low viral yield. We concluded that degradation of the miRNA H11 by VP55 attenuates viral replication.

In this study, we showed that the yields of viral proteins were reduced in cells infected with R5502 and the VP55-expressing HSV-1. Finally, the results suggested that, in cell cultures infected with R5502, the size of plaques-a direct measure of yields and cell-to-cell viral spreading-is also diminished. Early studies indicated that HSV-1 mutants lacking one or two origins are competent for lytic replication in cell culture (Polvino-Bodnar et al., 1987; Igarashi et al., 1993). OriL plays a role in *in vivo* replication or pathogenesis (Balliet and Schaffer, 2006). However, in this study, degradation of miRNA H11 by VP55 attenuated viral replication and restricted viral spreading from cell cultures. Thus, it is reasonable to suggest that the mutations in OriL altering pathogenicity exert their influence through miR-H11.

miR-H11 is homologous to one of the HSV-2 miRNA prediction termed T-4 by Cui et al. (2006) and Jurak et al. (2010). With regard to potential targets H11 to host cell factors, it cannot be excluded that the attenuation of viral replication through degradation of miR-H11 is attributed to the upregulation of host antiviral genes or downregulation of cellular genes which support viral replication. Lastly, degradation of unknown host miRNAs by VP55 may also contribute to the suppression of viral replication.

## Data Availability Statement

The datasets generated for this study can be found in the SRA repository. The SRA accession number is PRJNA613860 (https://www.ncbi.nlm.nih.gov/sra/PRJNA613860).

## Author Contributions

XC and GZ designed the study. WZ, XZ, and LW performed the research. WZ, XZ, XC, and GZ analyzed the data and wrote the manuscript.

## Conflict of Interest

The authors declare that the research was conducted in the absence of any commercial or financial relationships that could be construed as a potential conflict of interest.
