# Supplementary figures and images for: Degradation of Herpes Simplex Virus-1 Viral miRNA H11 by Vaccinia Virus Protein VP55 Attenuates Viral Replication
Source: Front Microbiol. 2020 Apr 23;11:717. doi: 10.3389/fmicb.2020.00717 (PMC7191008; doi:10.3389/fmicb.2020.00717)

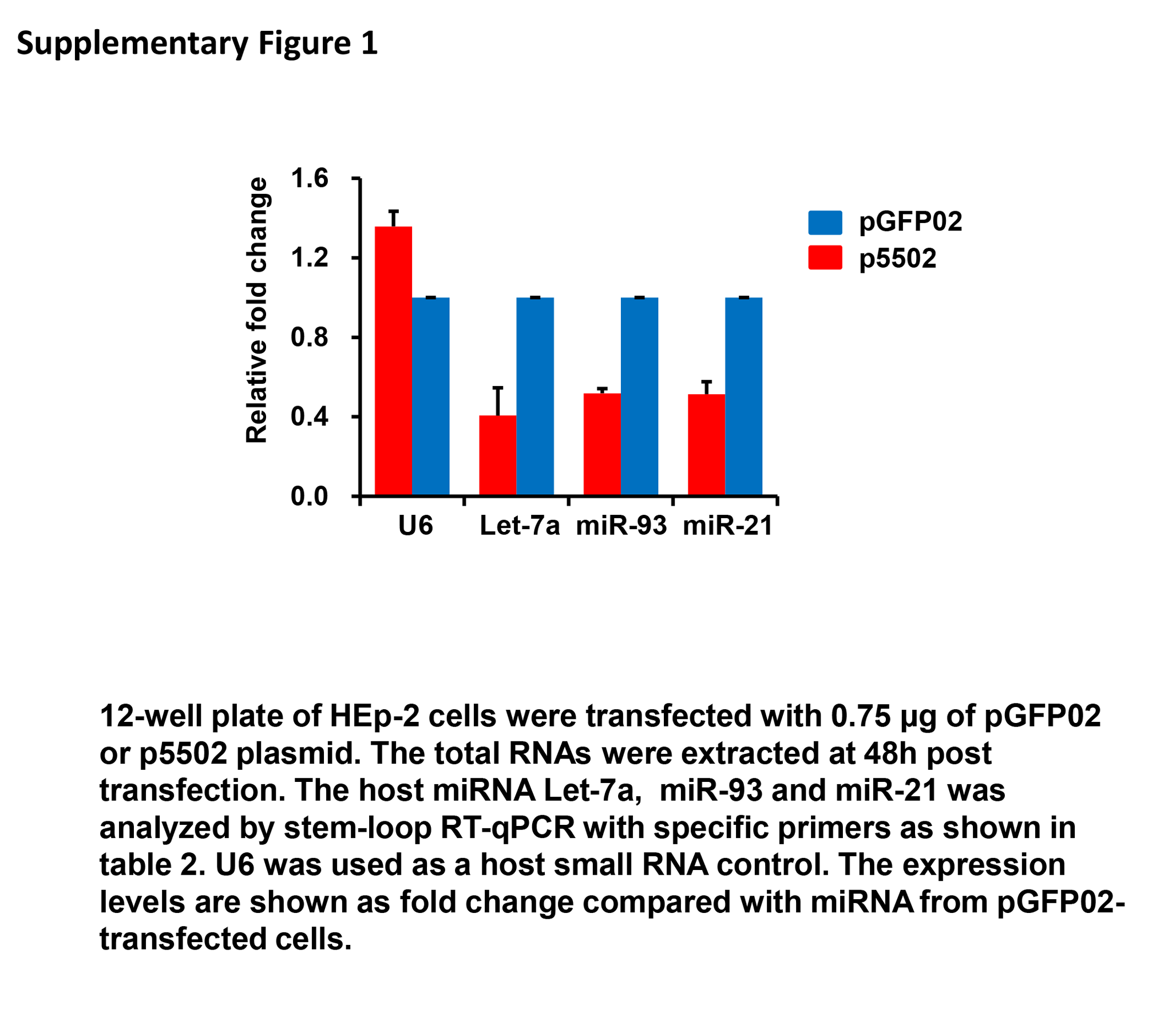

Supplement: Supplementary file 1 [file Image_1.TIF]
