# Supplementary material for: Degradation of Herpes Simplex Virus-1 Viral miRNA H11 by Vaccinia Virus Protein VP55 Attenuates Viral Replication
Source: Front Microbiol. 2020 Apr 23;11:717. doi: 10.3389/fmicb.2020.00717 (PMC7191008; doi:10.3389/fmicb.2020.00717)
Supplement: Supplementary file 2 [file Table_1.doc]

Supplementary Table 1. Sequences of miRNA primers

| **Gene** | **Forward primer** | **Reverse primer** | **Probe** |
| --- | --- | --- | --- |
| **U6** | 5'-CTCGCTTCGGCAGCACA-3' | 5'-AACGCTTCACGAATTTGCGT-3' | 5'-CTCTGTATCGTTCCAATTTTAGTAT-3' |
| **Let-7a** | 5'-GCCCGGTGAGGTAGTGGGTTGT-3' | 5'-CCAGTGCAGGGTCCGAGGTA-3' | 5'-CTGGATACGACAACTAT-3' |
| **miR-93** | 5'-CGGTGCAAAGTGCTGTTCG-3' | 5'-CCAGTGCAGGGTCCGAGGTA-3' | 5'-CTGGATACGACCTACCTGC-3' |
| **miR-21** | 5'-GCGTGCGCTAGCTTATCAGACTG-3' | 5'-CCAGTGCAGGGTCCGAGGTA-3' | 5'-CTGGATACGACTCAACATCA-3' |
| **18s rRNA** | 5'-CTCAACACGGGAAACCTCAC-3' | 5'-CGCTCCACCAACTAAGAACG-3' | 5'-CAGGATTGACAGATTGA-3' |
